# Bowel Necrosis in Leptospirosis: A Case Series of a Rare Complication

**DOI:** 10.1155/crdi/8189562

**Published:** 2025-11-14

**Authors:** Nalaka Herath, Sampath Hemachandra, Malaka Ranaweera, Asanka Sakalasooriya, Kosala Weerakoon, Shamila De Silva

**Affiliations:** ^1^Department of Nephrology, Teaching Hospital, Kurunegala, Sri Lanka; ^2^Department of Parasitology, Faculty of Medicine and Allied Sciences, Rajarata University, Anuradhapura, Sri Lanka; ^3^Department of Medicine, Faculty of Medicine, University of Kelaniya, Ragama, Sri Lanka

**Keywords:** acute kidney injury, bowel ischemia, leptospirosis, myocarditis, necrosis

## Abstract

**Background and Objectives:**

Leptospirosis, a globally prevalent zoonotic disease, exhibits diverse clinical manifestations, often with severe multiorgan involvement. Gastrointestinal complications are uncommon, but their potential severity and impact on patient outcomes warrant attention. We present two cases of severe leptospirosis complicated by terminal ileal and colonic necrosis.

**Patients and Methods:**

Two patients presented with acute febrile illness, severe myalgia, oliguria, and hypotension. Both had occupational exposure to paddy fields, a known risk factor for leptospirosis. Based on clinical presentation and laboratory findings (acute kidney injury, myocarditis, and acute severe pulmonary hemorrhagic syndrome), a diagnosis of severe leptospirosis was established. Development of proximal bowel obstruction and melena in the third week of illness indicated severe gastrointestinal involvement. Both patients received intensive care support, including broad-spectrum antibiotics, inotropes, and renal replacement therapy. One patient underwent exploratory laparotomy for bowel perforation. Despite aggressive management, both patients succumbed to the disease.

**Conclusions:**

Gastrointestinal complications, including bowel necrosis and perforation, can occur in severe leptospirosis. Early recognition and management of gastrointestinal symptoms are crucial. Further research is needed to understand the pathophysiology of this rare but fatal complication.

## 1. Introduction

Leptospirosis is the most common zoonotic disease across the globe caused by pathogenic spirochete *Leptospira interrogans* [1]. Human infection occurs through direct or indirect contact with urine of infected animals including pets, livestock, or wild/feral animals. Spread of infection is seasonal and occurs mostly during rainy seasons in the tropics [1, 2]. Sri Lanka is a tropical country reporting many cases of leptospirosis annually, with frequent outbreaks from different parts of the island [3].

Clinical presentations of leptospirosis range from a mild flu-like illness to multiorgan involvement. Organ involvement depends on the type and virulence of *Leptospira* and host susceptibility and varies widely, with hematological and renal involvement being the commonest [4]. Gastrointestinal involvement in leptospirosis is uncommon and includes pancreatitis, acalculous cholecystitis, and peritonitis [5–8]. We describe two rare cases of terminal ileal and proximal colonic necrosis following severe leptospirosis.

## 2. Case 1

A 55-year-old previously healthy man presented with fever, diarrhea, nausea, vomiting, myalgia, and dyspnea for three days after working in paddy fields. On admission, he was conscious, with low blood pressure (94/63 mmHg) and tachycardia (pulse rate 145 beats/min). There were bibasal lung crepitations, and urine output was only 10 mL over 4 hours. Admission blood tests indicated leucopenia and severe thrombocytopenia (Table 1).

After crystalloid fluid resuscitation noradrenaline was initiated to maintain blood pressure, intravenous ceftriaxone was commenced, and the patient was transferred to the medical intensive care unit (ICU). Due to severity of leptospirosis, intravenous methylprednisolone was administered for 3 days, and multiple platelet transfusions were given to correct thrombocytopenia. As oliguria persisted, intermittent hemodialysis was initiated. Dopamine was added due to persistent hypotension.

**Figure 1:**
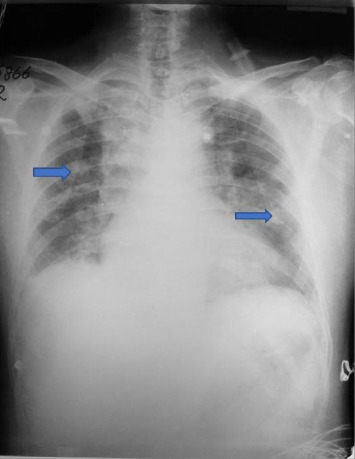
Case 1: Chest radiograph showing bilateral confluent alveolar lesions (arrows) suggestive of severe pulmonary hemorrhagic syndrome.

Chest radiograph indicated severe pulmonary hemorrhagic syndrome (Figure 1). Echocardiogram showed hypokinetic anterior wall motion and moderate mitral and tricuspid regurgitation. The patient was electively intubated and ventilated due to respiratory acidosis and hypoxia. Despite treatment, thrombocytopenia and abnormal liver function tests persisted (Table 1). Therapeutic plasma exchange was performed, and meropenem was commenced.

By Day 8, the patient was stable and afebrile, allowing for gradual withdrawal of inotropes. Leptospira microscopic agglutination test (MAT) became positive with a high titer. Blood picture showed low-grade hemolysis with a few red cell fragments.

**Figure 2:**
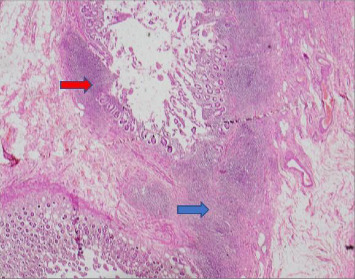
Case 1: Histopathological appearance of intestinal biopsies confirming extensive bowel necrosis (red arrow) from terminal ileum to hepatic flexure with perforation and abscess formation (blue arrow) related to the posterior wall of ascending colon (hematoxylin and eosin stain).

On Day 21, the patient experienced fresh rectal bleeding. Upper gastroesophageal endoscopy revealed multiple gastric erosions, and colonoscopy revealed features of ischemic colitis. Blood and fresh frozen plasma were transfused, and intravenous proton pump inhibitor infusion was commenced. Exploratory laparotomy revealed extensive bowel necrosis from terminal ileum to the hepatic flexure with perforation and abscess formation of posterior wall of the ascending colon. Histopathology of affected areas confirmed these changes (Figure 2). There was no evidence of vasculitis in the rest of the peritoneal viscera. Standard right hemicolectomy and elective tracheostomy were performed.

On postoperative Day 1, the patient became unstable with hypotension and severe acidosis. Intact exteriorized bowel ends which had looked healthy at surgery showed features of ischemic necrosis. Since the patient's condition was unstable, further surgical exploration was abandoned. He succumbed on Day 27 of illness due to multiorgan failure.

## 3. Case 2

A 52-year-old man with Type 2 diabetes presented with fever, arthralgia, and myalgia for three days after working in paddy fields. On admission, he was febrile, icteric, dyspneic, and hypotensive (blood pressure 93/60 mmHg), with bibasal lung crepitations. He had not urinated for over 6 hours.

Initial tests indicated neutrophilic leukocytosis, severe thrombocytopenia, and elevated liver enzymes and serum creatinine (Table 2). Arterial blood gas analysis showed hypoxemia and metabolic acidosis with a lactate level of 7.1 mmol/L. Chest radiograph suggested severe pulmonary hemorrhagic syndrome. Leptospira MAT was positive at a titer of 1 : 640.

After fluid resuscitation noradrenaline was initiated, the patient was transferred to the ICU. He was electively intubated due to severe hypoxemia. Intravenous ceftriaxone was commenced. Renal replacement therapy by continuous venovenous hemodiafiltration was commenced, and multiple platelet concentrates were given to correct thrombocytopenia. Once stabilized, intermittent hemodialysis was initiated.

**Figure 3:**
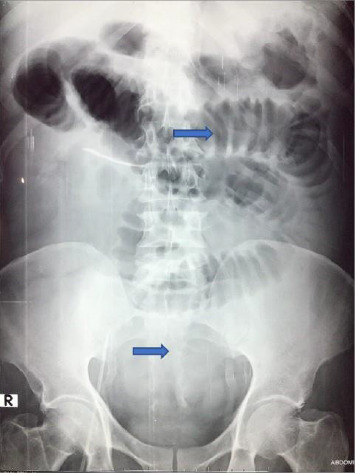
Case 2: Radiograph of abdomen (anteroposterior view) showing dilated multiple small bowel loops in central abdomen (arrow) with collapsed large bowel without free gas in peritoneal cavity, suggestive of small intestinal obstruction.

On Day 13, the patient developed blood-stained nasogastric aspirate and significant anemia. Upper gastrointestinal endoscopy revealed gastric erosions. Anemia was corrected with multiple blood transfusions. The patient was gradually improving by this time with no fever and minimum ventilator support until Day 15, when abdominal tenderness and sluggish bowel sounds were detected. There was intolerance of nasogastric feeds and melena. The abdomen was distended, and an anteroposterior radiograph (Figure 3) showed dilated multiple small bowel loops, a collapsed large bowel but no free gas in the peritoneal cavity. An ultrasound scan confirmed small bowel obstruction. The patient was hemodynamically unstable for contrast-enhanced abdominal computed tomography scan or for any surgical intervention. He succumbed on Day 21 of illness.

## 4. Discussion

Bowel perforation due to leptospirosis is a rare complication [5–9]. We describe two fatal cases of leptospirosis associated with bowel necrosis causing perforation. The clinical presentation of these two patients was complex, as they both developed multiple organ-related complications. Both patients presented with hypotension requiring inotropes and acute kidney injury requiring renal replacement therapy for more than 2 weeks. Both patients developed severe pulmonary hemorrhagic syndrome and acute myocarditis. All complications were managed in the ICU, and both patients were slowly recovering when bowel ischemia developed as a late complication in the third week of illness.

Colorectal manifestations are rare in leptospirosis [5, 9] and can be easily missed due to the complexity of cases. Gastrointestinal involvement is diverse, ranging from ulcers to necrosis and perforation with peritoneal sepsis [5–9]. These severe complications are attributed to the inherent pathogenicity of different strains as well as host immunopathological responses, while the exact pathophysiology is not fully described. Bowel ischemia and perforation may be due to complex interactions of various tissue-injuring factors, namely, hemolysis, endotoxins, and lipase [5–8]. Vasculitis of capillaries may contribute to ischemia and necrosis [4, 5]. Hemorrhagic complications may also occur secondary to disease activity, coagulopathy, and thrombocytopenia [10, 11]. Prolonged hypotension and the use of inotropes may contribute to ongoing ischemic necrosis.

Bowel ischemia and necrosis due to leptospirosis are not a common case for a surgeon since it is an extremely rare condition. Especially in a healthy individual without prior risk factors for mesenteric ischemia, the diagnosis itself is challenging. Since no real data are available to predict which leptospirosis patient would develop gut ischemia, to decide early surgical intervention is extremely difficult.

Mesenteric vasculitis, a rare condition, can be challenging to diagnose due to nonspecific symptoms and the rarity of the disease. However, it might be useful to do CT angiogram/CECT abdomen and pelvis, in patients with presentations like these two cases, when such patients are not improving with conventional treatment for leptospirosis and are showing unorthodox clinical features such as bowel obstruction or gut ischemia.

Both our patients had lower gastrointestinal bleeding indicative of severe terminal ileal and colonic involvement with leptospirosis during the convalescent period. This could be related to *Leptospira* antigen-associated vasculoendothelial damage. [12]. This may also be due to prolonged hypotension causing bowel ischemia, as the third week of illness is too late for direct invasion of *Leptospira* and immune-related complications. Although watershed areas of the intestines are the splenic flexure and rectosigmoid junction, the first patient had ischemic necrosis in the terminal ileum, cecum, and proximal colon, which are generally considered to have good blood supply.

Bowel necrosis was confirmed in Case 1, but it was not possible to histologically confirm the same in Case 2. This is a limitation of this case series. However, bowel necrosis was strongly suspected in Case 2 based on clinical deterioration and signs of intestinal obstruction.

## 5. Conclusion

Bowel necrosis following severe leptospirosis carries a poor prognosis. Specific mechanisms of pathogenesis are unclear. Severe gastrointestinal complications may be attributed to the inherent pathogenicity of different strains or host immunopathological responses. Prolonged hypotension and the use of inotropes may contribute to ongoing bowel ischemia and necrosis.

## Figures and Tables

**Table 1:** Laboratory findings of Case 1 at different time points of management.

Description	Reference range	Day 4	Day 5	Day 6	Day 8	Day 14	Day 18	Day 21	Day 24
White cell count (× 10^3^/μL)	4.5–11	0.92	3.7	21.6	17.3	11.9	10.5	12.4	14.47
Hemoglobin (g/dL)	13–17.5	11.5	11.7	10.1	10.5	10	8.7	6.9	8.8
Platelet count (× 10^9^/L)	150–450	28	18	36	36	52	162	107	22
Serum creatinine (μmol/L)	60–120	290	272	257	488	316	284	355	144
Blood urea (mg/dL)	7–20	90	82	76	208	130	129	166	71
CRP (mg/L)	< 10	247	240	275	95	252		95.2	203
Troponin I (ng/mL)	< 0.04	> 1.8		> 1.8	2.66		1–1.5		
pH	7.35–7.45	7.15	7.2	7.37					7.11
Bicarbonate (mmol/L)	22–29	12	15	20					12
Lactate (mmol/L)	0.5–2.2	3.2	3.5	4.9					5.8
SGOT (U/L)	8–45	254		365	434	46	45	37	119
SGPT (U/L)	7–56	105		127	352	23	24	27	44.9
INR	0.8–1.2	1.02	1.3	2.03	1.6	1.3	1.26	1.35	2.1
APTT (seconds)	25–35		36		13			30.4	44
Serum bilirubin (mg/dL)	0.1–1.2	30		38	32	40	28.9	21.7	42
LDH (U/L)	135–225			806	1548	472	435	372	663
CPK (U/L)	55–170			1339	2705				

**Table 2:** Laboratory findings of Case 2 at different time points of management.

Description	Reference range	Day 4	Day 5	Day 7	Day 13	Day 15	Day 17	Day 21
White cell count (× 10^3^/μL)	4.5–11	4.27	13.4	9	23	23	17	3.9
Hemoglobin (g/dL)	13–17.5	12.7	13.9	11	9.8	9.9	8.8	9.5
Platelet count (× 10^9^/L)	150–450	50	17	46	62	87	66	44
Serum creatinine (μmol/L)	60–120	265	241	353	477	325	334	377
Blood urea (mg/dL)	7–20	88.1	80	247	253	175	196	226
CRP (mg/L)	< 10	475	448	421	250	363	267	345
Troponin I (ng/mL)	< 0.04		7.23	12.2			0.23	
pH	7.35–7.45	7.34	7.36	7.43				
Bicarbonate (mmol/L)	22–29	9.3	22.7	21.7				
Lactate (mmol/L)	0.5–2.2	11.3	7.1	2.4				
SGOT (U/L)	8–45	167	171	161	120	97	65	46
SGPT (U/L)	7–56	63	68	50	16	20	14	6
INR	0.8–1.2		1.1	1.3				1
Serum bilirubin (mg/dL)	0.1–1.2	39.6	55	125	161	169	140	262

## Data Availability

The clinical information, records, and images used in the preparation of this manuscript are available from the corresponding author on request.
